# Evaluation of Immunogenicity and Protective Efficacy Elicited by *Mycobacterium bovis* BCG Overexpressing Ag85A Protein against *Mycobacterium tuberculosis* Aerosol Infection

**DOI:** 10.3389/fcimb.2016.00003

**Published:** 2016-01-28

**Authors:** Zheng Zhong Xu, Xiang Chen, Ting Hu, Chuang Meng, Xiao Bo Wang, Yan Rao, Xiao Ming Zhang, Yue Lan Yin, Zhi Ming Pan, Xin An Jiao

**Affiliations:** ^1^Jiangsu Key Laboratory of Zoonosis, Jiangsu Co-Innovation Center for Prevention and Control of Important Animal Infectious Diseases and Zoonoses, Yangzhou UniversityYangzhou, China; ^2^ABSL-3 Lab, Wuhan UniversityWuhan, China; ^3^Unit of Innate Defense and Immune Modulation, Institute Pasteur of Shanghai, Chinese Academy of SciencesShanghai, China

**Keywords:** *Mycobacterium tuberculosis*, recombinant BCG, Ag85A, immunogenicity, protective efficacy

## Abstract

*Mycobacterium bovis* bacillus Calmette-Guérin (BCG) is currently the only vaccine available for preventing tuberculosis (TB), however, BCG has varying success in preventing pulmonary TB. In this study, a recombinant BCG (rBCG::Ag85A) strain overexpressing the immunodominant Ag85A antigen was constructed, and its immunogenicity and protective efficacy were evaluated. Our results indicated that the Ag85A protein was successfully overexpressed in rBCG::Ag85A, and the Ag85A peptide–MHC complexes on draining lymph node dendritic cells of C57BL/6 mice infected with rBCG::Ag85A were detectable 4 h post-infection. The C57BL/6 mice infected with this strain had stronger antigen-specific interferon-gamma (IFN-γ) responses and higher antibody titers than those immunized with BCG, and the protective experiments showed that rBCG::Ag85A can enhance protection against *Mycobacterium tuberculosis* (*M. tuberculosis*) H37Rv infection compared to the BCG vaccine alone. Our results demonstrate the potential of rBCG::Ag85A as a candidate vaccine against TB.

## Introduction

Tuberculosis (TB) is caused by the intracellular pathogen *Mycobacterium tuberculosis* is (*M. tuberculosis*), and is responsible for about 2 million deaths each year and an estimated 9 million new cases annually (Rappuoli, 2014). Despite a TB vaccine being available for more than 90 years, TB remains a major global health issue (Rappuoli, 2014). The bacillus Calmette-Guérin (BCG) vaccine is effective against miliary and meningeal TB in children, but provides variable protection against pulmonary TB in adults (Andersen and Doherty, 2005). Therefore, alternative vaccines or supplements to the BCG vaccine are needed.

However, the development of novel vaccines outperforming BCG is difficult. Recently, 12 new TB vaccines have entered clinical trials, which are focused on two goals: (1) improve the existing vaccine by replacing BCG with attenuated bacterial strains derived from BCG or *M. tuberculosis*, for example, VPM1002 (rBCGΔureC::hly) was an attenuated strain, which was safe and immunogenic for B-cell and T-cell responses, and it is the most advanced recombinant vaccine in clinical trial (Grode et al., 2013), while rBCG30 strain was obtained by engineering BCG to overexpress Ag85B to induce better immune response (Gillis et al., 2014), (2) improve the TB vaccine by boosting BCG induced immunity using protein-based vaccines delivered in the presence of adjuvants or with viral vectors. For example, the H1 vaccine comprises a fusion protein of Ag85B-ESAT6 in the adjuvant IC31, while others were delivered via DNA or viral vectors, such as MVA85A and Ad5HUAG85A (Rappuoli and Aderem, 2011; Knudsen et al., 2014).

One of the most popular antigens used to generate TB vaccines is the TB antigen 85A (Ag85A). Ag85A comprises a major fraction of the secreted proteins in *M. tuberculosis* and *Mycobacterium bovis* BCG culture filtrate, and belongs to the Ag85 complex, a 30- to 32-kDa family of three proteins (Ag85A, Ag85B, and Ag85C; Wiker and Harboe, 1992; Belisle et al., 1997), all of which exhibit mycolyltransferase activity. These proteins are encoded by three paralogous genes located in distinct regions of the bacterial genome (Content et al., 1991). Ag85A can induce strong T-cell proliferation and IFN-γ production in healthy individuals infected with *M. tuberculosis* and in *M. bovis* BCG-vaccinated mice (D'souza et al., 2003). Because this antigen induces protective immune responses, it is among the most promising candidates for use in future development of tuberculosis vaccines. MVA85A is a modified vaccinia virus Ankara (MVA): a live-attenuated poxvirus vector expressing Ag85A. This virus induces strong CD4^+^ T cell responses in animals and humans, and provides enhanced protection in BCG-primed MVA85A-boosted animals challenged with *M. tuberculosis* (Verreck et al., 2009). However, in a recent trial, MVA85A was given to infants as a BCG booster, but the presence of MVA85A protein did not protect against TB infection better than the BCG immunization alone (Tameris et al., 2013; Harris et al., 2014). Ad5HUAG85A is human Ad5 expressing Ag85A, and the Ad induces CD8^+^T cell responses, but the pre-existing antibodies may cause the elimination thus reducing the vaccine efficacy (Kaufmann et al., 2014).

While adding MVA85A or Ad5HUAG85A as the booster to the BCG vaccine exhibited no significant improvement in vaccine efficacy, there is no doubt that the Ag85A antigen itself is able to induce protection, so an approach via overexpressing the tuberculosis antigen Ag85A in attenuated BCG strains may have great promise in TB vaccine development. In this study, we generated a recombinant BCG strain that overexpresses the immunodominant Ag85A antigen, and evaluated its immunogenicity and protective efficacy in mice challenged with aerosolized *M. tuberculosis*, to assess its potential as a candidate vaccine against TB.

## Materials and methods

### Experimental animals

Six-week-old female C57BL/6 mice were purchased from VITAL RIVER (Beijing, China). Mice were housed, handled, and immunized at the animal biosafety facilities and all procedures were approved by the institutional animal experimental committee of Yangzhou University. All *M. tuberculosis* H37Rv challenge experiments were performed in the Animal Biosafety Level 3 (ABSL-3) facility of Wuhan University.

### Bacterial strains and cell culture

The *Escherichia coli* strain DH5α was used for cloning and grown in Luria broth (LB). *M. bovis* BCG Pasteur 1173P2 and rBCG were grown in Middle brook 7H9 medium (Difco, MI, USA) supplemented with 0.05% Tween 80 and 10% acid–albumin–dextrose–catalase complex (ADC), or on solid Middle brook 7H10 medium (Difco) supplemented with oleic acid–albumin–dextrose–catalase complex (OADC). Kanamycin was added when required (final concentration 25 μg/ml). The Ag85A epitope-specific (241–260) T cell hybridoma (DE10) was a gift from Dr. Claude Leclerc (Institut Pasteur, Paris; Johansen et al., 2011).

### Construction of recombinant BCG

The gene fragment, *fbpA*, was PCR amplified using *M. bovis* BCG Pasteur 1173P2 chromosomal DNA as a template. The forward primer (5′-TA GGA TCC ATG CAG CTT GTT GAC AG-3′) contained a *Bam*HI restriction site and the reverse primer (5′-TA GAA TTC GTT GTG TCT GTT CGG AGC-3′) contained an *Eco*RI restriction site. The resulting 1050 bp fragment was ligated to the T-cloning site of a pCR2.1 vector (Invitrogen, Carlsbad, CA, USA), isolated by digestion with *Bam*HI and *Eco*RI, and ligated into the shuttle plasmid pMV261 to generate pMV261–*fbpA*. The recombinant shuttle plasmid was transformed into a wild type BCG strain by electroporation as described previously (Wang et al., 2009). The BCG-transformed bacterial cells were incubated on 7H10 solid media (supplemented with 25 μg/ml kanamycin) and grown at 37°C for 2–3 weeks. Transformants were selected and confirmed by PCR using forward (5′-GAG GAA TCA CTT CGC AAT GG-3′) and reverse primers (5′-GCC TTT CGT TTT ATT TGA TGC C-3′) that lie 5′ and 3′ of pMV261 MCS.

### Western blot analysis

BCG-positive transformants were grown in Middle brook 7H9 medium containing 25 μg/ml of kanamycin. After 2–3 weeks, protein expression was induced by incubating the cultures at 45°C for 2 h. rBCG cells were centrifuged at 8000 rpm for 10 min, and cell lysate was separated on a 12% SDS-PAGE gel. Proteins were then transferred to a PVDF membrane, and the membrane was blocked with 2% bovine serum albumin (BSA) in phosphate buffered saline (PBS) at room temperature for 2 h. After blocking, the membrane was washed three times with PBS containing 0.05% Tween 20 (PBST). The membrane was then incubated with an anti-Ag85A monoclonal antibody (mAb) 6B5 (1:1000; preserved at Jiangsu Key Laboratory of Zoonosis) at room temperature for 1 h. After three consecutive washes with PBST, the membrane was incubated with a horseradish peroxidase (HRP)-labeled goat anti-mouse secondary antibody (1:2000; Sigma, St. Louis, MO, USA). Finally, the membrane was developed with 3,3′-diaminobenzidine tetrahydrochloride (Sigma, USA) and visualized using X-ray film.

### Indirect enzyme-linked immunosorbent assays (ELISAs)

ELISA plates (Nunc, Denmark) were treated with 5% glutaraldehyde (100 μl/well) and incubated at 37°C for 2 h. The plates were washed three times with PBST, then coated with 1 × 10^6^ colony-forming units (CFU) of BCG or rBCG::Ag85A bacteria (100 μl/well) overnight at 56°C, after three washes with PBST the plates were blocked with PBS containing 2% BSA at 37°C for 2 h. The plates were washed three more times with PBST, and mAb 6B5 was added at two-fold serial dilutions (beginning at a 1:100 dilution) and incubated at 37°C for 2 h. HRP-labeled goat anti-mouse IgG (1:8000 dilution; Sigma-Aldrich, USA) was added to each well (100 μl/well) and incubated at 37°C for 1 h. The TMB substrate was then added to the plates and incubated for 10 min, after which the plates were read at 450 nm.

### Immunizations and challenge

Groups of C57BL/6 mice (10 per group) were immunized subcutaneously (s.c) with 5 × 10^6^ CFUs of either BCG or rBCG::Ag85A suspended in 100 μl PBS. Unvaccinated mice, which served as a control, received an equal volume of PBS. At 6 weeks post-vaccination, mice were challenged with aerosolized *M. tuberculosis* H37Rv with Glas-Col chamber as described previously (Zhang et al., 2011), during which time approximately 200 bacteria were deposited in the lungs of each animal.

### Antigen presentation assays

C57/BL6 mice were injected subcutaneously with 5 × 10^6^ CFU of BCG or rBCG::Ag85A bacteria, and their draining lymph nodes were removed at 0, 4, 24, and 48 h post-injection, respectively, and perfused with 400 U/ml of collagenase type IV (Invitrogen) containing 50 μg/ml of DNase I (Invitrogen). Single-cell suspensions were prepared from the isolated lymph nodes and dendritic cells (DCs) were sorted with an autoMACS instrument (MiltenyiBiotec, Germany) using anti-CD11c microbeads (MiltenyiBiotec, Germany), leading to a CD11c^+^ positive cell sample >90% purity. For the *ex vivo* antigen presentation assay, 1 × 10^5^ isolated DCs were added to 96-well microplates, then 1 × 10^5^ DE10 T cell hybridomas were added to the antigen presenting cells, and incubated at 37°C in a 5% CO_2_ atmosphere for 24 h. The supernatants were harvested, frozen and tested for IL-2 production using a sandwich ELISA (BD Biosciences, USA).

### Cytokine production

BCG and rBCG::Ag85A-vaccinated mice were sacrificed 6 weeks post-immunization, and their spleens and draining lymph nodes were removed aseptically in RPMI-1640 medium supplemented with 10% fetal calf serum, 100 g/ml streptomycin, and 100 IU/ml penicillin. The single-cell suspensions were prepared using Histopaque 1083 (Sigma, USA), and then the cells were added to 96-well plates containing RPMI-1640 medium (1 × 10^6^ cells/well in 200 μl media). Cells were stimulated with 10 μg/ml of Ag85A peptide, 10 μg/ml of Ag85A protein, or 5 μg/ml of bovine purified protein derivative (PPD, Prionics, Switzerland). The cells were incubated at 37°C in a 5% CO_2_ atmosphere, and the supernatants were harvested 48 h post-stimulation, frozen, and tested for IFN-γ and IL-4 production by sandwich ELISA (BD Biosciences, USA).

### Ag85A-specific IgG ELISA assays

C57BL/6 mouse sera were collected from the various groups of mice for antibody detection. ELISA plates (Nunc) were coated with Ag85A (5 μg/ml, 100 μl/well) overnight at 4°C. The plates were washed three times with PBST, and blocked with 2% BSA in PBS for 2 h at 37°C. Samples were washed three additional times, and treated with serum samples at two-fold serial dilutions (beginning at 1:500 dilution) for 2 h at 37°C. HRP-labeled goat anti-mouse IgG (Sigma-Aldrich), HRP-labeled goat anti-mouse IgG1 (Invitrogen), and HRP-labeled goat anti-mouse IgG2b (Southern Biotec, Birmingham, AL, USA) were used at 1:8000 dilutions; 100 μl of the serially diluted antibodies were added individually to the wells of each plate, and the plates were incubated at 37°C for 1 h. The substrate TMB was added and incubated for 10 min, and the plates were read at 450 nm.

### CFU measurements

Mice were sacrificed 6 weeks post *M. tuberculosis* challenge, and their lungs and spleens were removed aseptically. For enumeration of actual CFU, a portion of spleens and lungs was weighed and homogenized, and the bacterial load was determined by plating 10-fold dilutions of the tissue homogenate on 7H10 Middle brook agar plates. Plates were incubated at 37°C for at least 3 weeks, and colonies were counted and expressed as log10 CFU per organ.

### Histopathological analysis

For histopathological study, lung and spleen tissues were fixed in 10% formalin and embedded in paraffin. Sections measuring approximately 4–6 μm were prepared and used for acid-fast staining and hematoxylin and eosin (HE) staining. Histopathological evaluation and scoring were performed by a single pathologist unaware of the treatment groups for at least twice to verify the reproducibility of the observations.

### Statistical analysis

All data are expressed as mean ± SEM. Statistical analysis was performed using a Student's *t*-test. A value of *P* < 0.05 was considered statistically significant.

## Results

### Construction and isolation of rBCG::Ag85A bacteria

The gene encoding Ag85A was amplified (Figure S1A) and cloned into the *E. coli*–*M. tuberculosis* shuttle vector, pMV261. The recombinant vector pMV261–*fbpA* (Figure S1B) was confirmed by sequencing, then transformed into the BCG Pasteur 1173P2 strain. Candidate rBCG::Ag85A clones were isolated and verified by PCR amplification, confirming that the recombinant vector pMV261–*fbpA* had been successfully transformed into the rBCG::Ag85A strain (Figure S1C).

### Confirmation of Ag85A overexpression by western blot analysis

**Figure 1:**
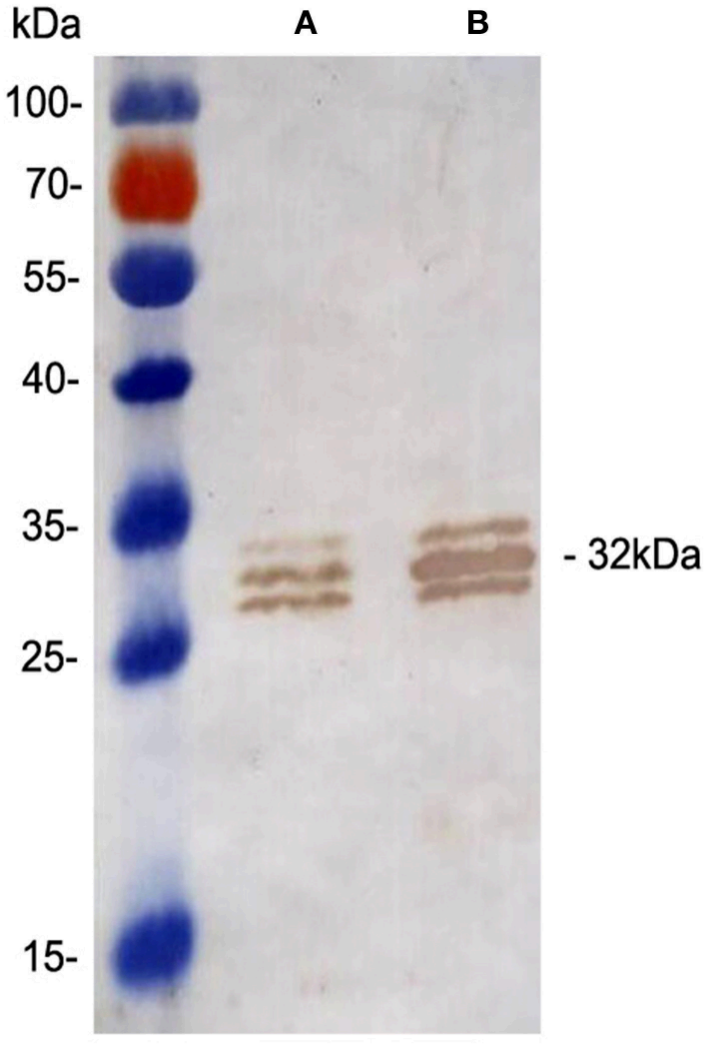
**Confirmation of Ag85A expression by Western blot analysis**. Western blot analysis of the 31 kDa Ag85A in BCG **(A)** and rBCG::Ag85A **(B)**. Ag85A protein levels increased two-fold in rBCG::Ag85A. Quantitative analyses of the protein bands were performed using Image J Software (National Institutes of Health, Maryland, USA).

Overexpression of the Ag85A protein in the rBCG::Ag85A strain was confirmed by western blotting using the anti-Ag85A mAb, 6B5. The untransformed BCG strain was used as a control. The results demonstrate that the 31 kDa Ag85A protein levels are increased two-fold in rBCG::Ag85A compared with those of BCG (Figure 1), while the 30 and 31.5 kDa proteins represent Ag85B and Ag85C, respectively.

### Evaluation of Ag85A expression by indirect ELISA

**Figure 2:**
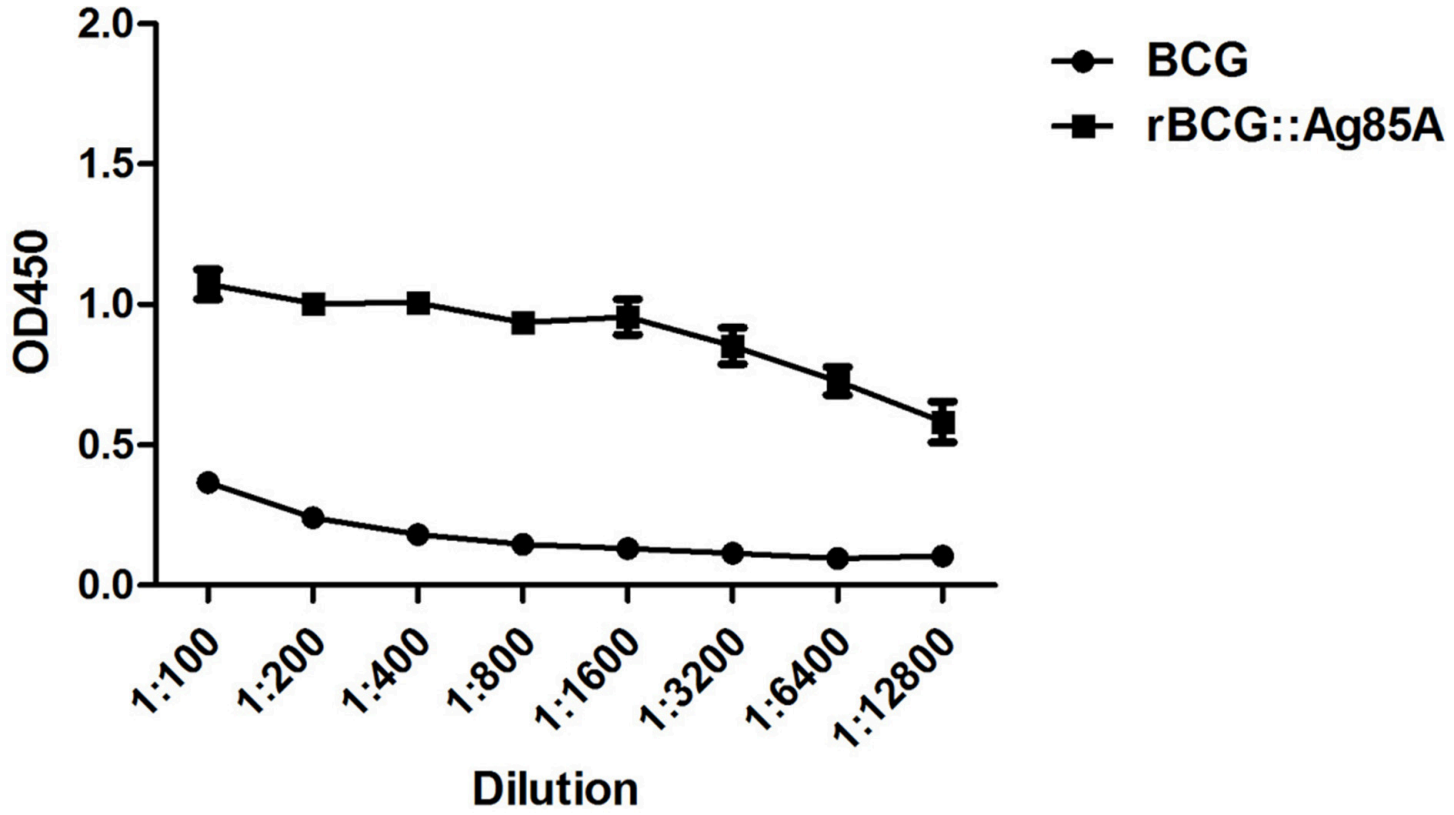
**Identification of Ag85A expression by indirect ELISA**. An ELISA plate coated with equal amounts of BCG and rBCG::Ag85A bacteria was probed for Ag85A expression with serially diluted mAb 6B5. Expression of Ag85A by rBCG::Ag85A was significantly higher than that by BCG. Data depicted are the mean values ± SEM. Statistical significance was determined by a Student's *t*-test.

To confirm the overexpression of Ag85A in rBCG::Ag85A, plates coated with equal amounts of BCG and rBCG::Ag85A bacteria were used to assess Ag85A expression by indirect ELISA using serially diluted 6B5 mAb. The optical density (OD) of rBCG Ag85A was significantly higher than that of BCG Ag85A (Figure 2), confirming that the Ag85A protein was successfully overexpressed in rBCG::Ag85A.

### Antigen presenting assays

**Figure 3:**
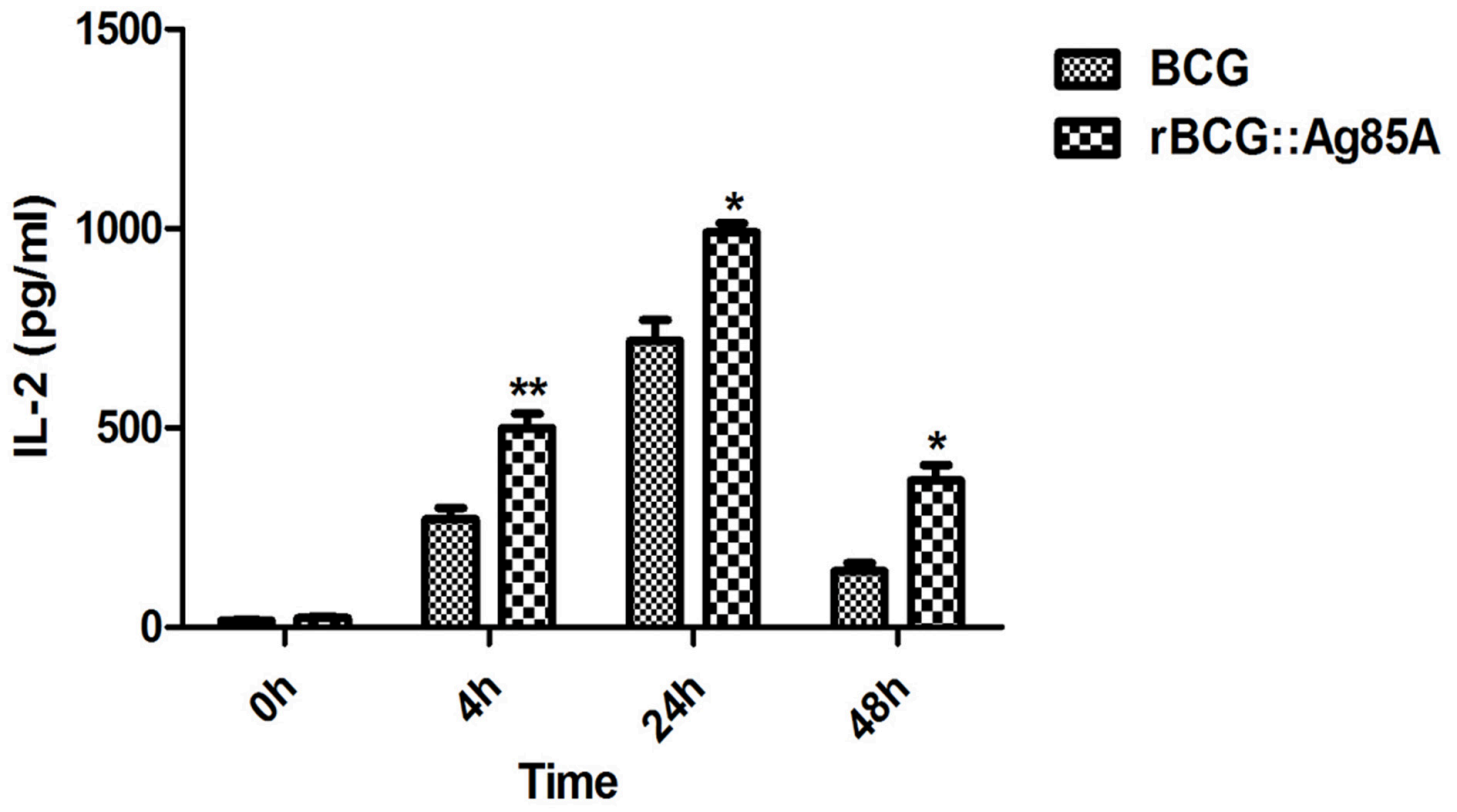
***Ex vivo* detection of antigen-presentation following rBCG::Ag85A infection**. After s.c. administration of rBCG::Ag85A, DCs from mouse lymph nodes were analyzed for their ability to stimulate the DE10 T hybridoma by probing for Ag85A peptide presentation in MHC complexes. Ag85A-M was detected 4 h post-rBCG infection. rBCG::Ag85A induced higher levels of Ag85A-specific antigen-presenting activity in mice compared with those immunized with BCG. Data depicted are the mean values ± SEM. Statistical significance was determined by a Student's *t*-test (^*^P < 0.05, ^**^P < 0.01).

Next, we investigated the antigen-presenting activity of the cells *ex vivo* by harvesting the draining lymph nodes from rBCG::Ag85A and BCG infected mice. After s.c. administration of rBCG::Ag85A, we isolated DCs from the lymph nodes and monitored their capacity to stimulate the DE10 T hybridoma, which is specific for a dominant Ag85A peptide. Under these conditions, the *in vivo* formation of Ag85A peptide–MHC complexes on DCs from BCG-infected mice was detected *ex vivo* by T cell hybridoma stimulation. Ag85A peptide–MHC complexes on the isolated DCs were detected as early as 4 h post rBCG::Ag85A immunization (Figure 3). rBCG::Ag85A immunization also induced a higher level of Ag85A-specific antigen-presenting activity than BCG alone.

### Cytokine responses

**Figure 4:**
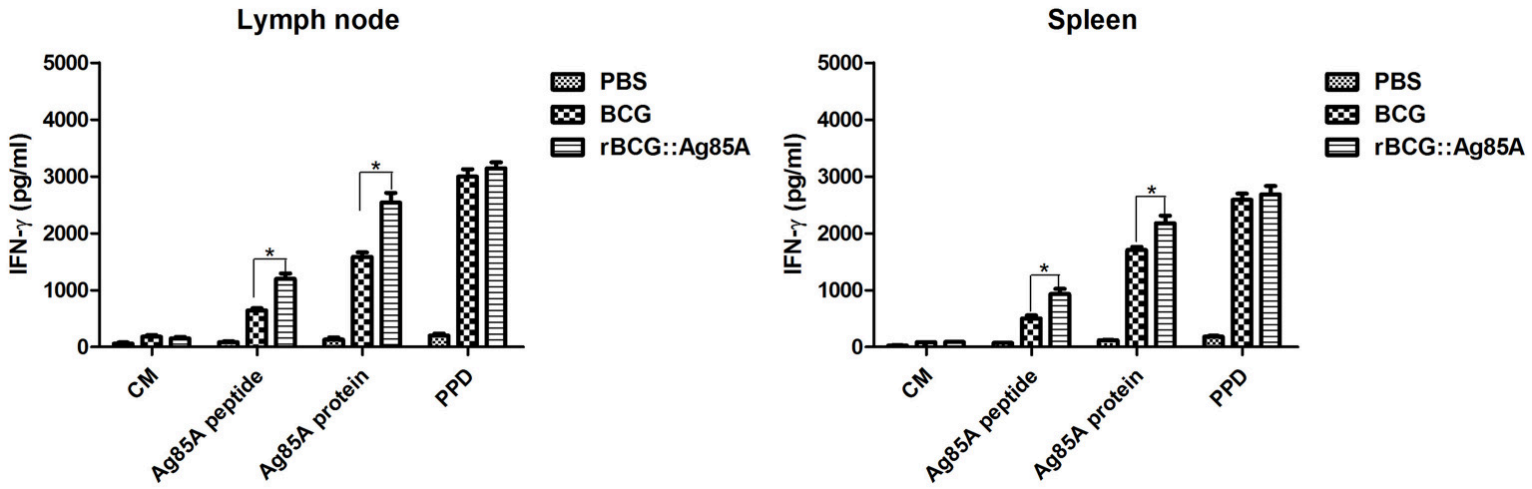
**Antigen-specific cellular immune responses following rBCG::Ag85A infection**. IFN-γ levels in culture supernatants of lymphocytes stimulated *in vitro* with either culture medium (CM), Ag85A peptide, purified Ag85A protein, or PPD were detected by sandwich ELISA. Lymphocytes from draining lymph nodes (left) or spleens (right) from the PBS control group produced very low levels of IFN-γ; higher levels of IFN-γ were found in the rBCG::Ag85A-immunized mice compared with those of the BCG-immunized mice. Data depicted represent the mean values ± SEM. Statistical significance was determined by a Student's *t*-test (^*^P < 0.05).

To evaluate the T cell immune responses primed by immunization with rBCG::Ag85A and BCG bacteria, we monitored IFN-γ cytokine levels in the culture supernatants of lymphocytes stimulated with either Ag85A peptide, purified Ag85A protein, or PPD antigens by ELISA. Lymphocytes from the draining lymph nodes and spleens from the PBS control group produced very low IFN-γ levels. The highest levels of IFN-γ production were observed in culture supernatants of lymphocytes derived from rBCG::Ag85A-immunized mice (Figure 4). In contrast, IL-4 production by all samples was below the detection limits of the assay (data not show). These results indicate that rBCG::Ag85A can enhance Th1 cytokine secretion.

### Antigen-specific humoral responses

**Figure 5:**
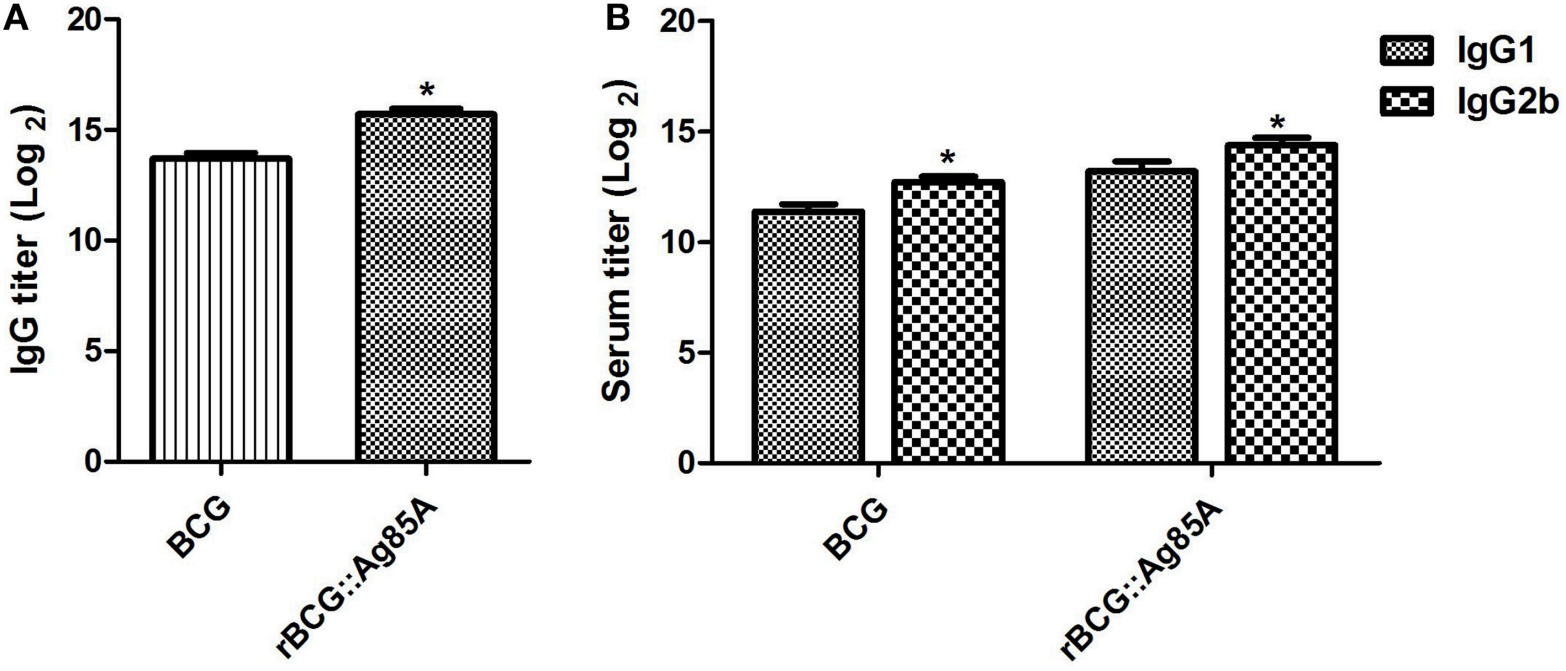
**Antigen-specific antibody responses following immunization with rBCG::Ag85A**. Mice immunized with rBCG::Ag85A had higher levels of Ag85A-specific IgG than those immunized with BCG **(A)**. The IgG2b to IgG1 ratio in the sera of mice immunized with rBCG::Ag85A was higher than that of BCG **(B)**. Data depicted represent the mean values ± SEM. Statistical significance was determined by a Student's *t*-test (^*^P < 0.05).

The specific antibody responses induced by BCG and rBCG::Ag85A strains were evaluated by ELISA, and IgG, IgG1, and IgG2b titers were calculated for the sera of mice sacrificed 4 weeks post-immunization. Mice immunized with rBCG::Ag85A exhibited higher levels of Ag85A-specific IgG than those immunized with BCG (Figure 5A), suggesting that rBCG::Ag85A induced a more efficient humoral response in mice than that induced by BCG alone. Furthermore, we analyzed titers of IgG1 and IgG2b, and found that the titers of IgG2b in the sera of rBCG::Ag85A-immunized mice were higher than IgG1 (Figure 5B). These results indicate that rBCG::Ag85A induced strong humoral and Th1 immune responses in the C57BL/ 6 mice.

### Determination of bacterial loads

**Figure 6:**
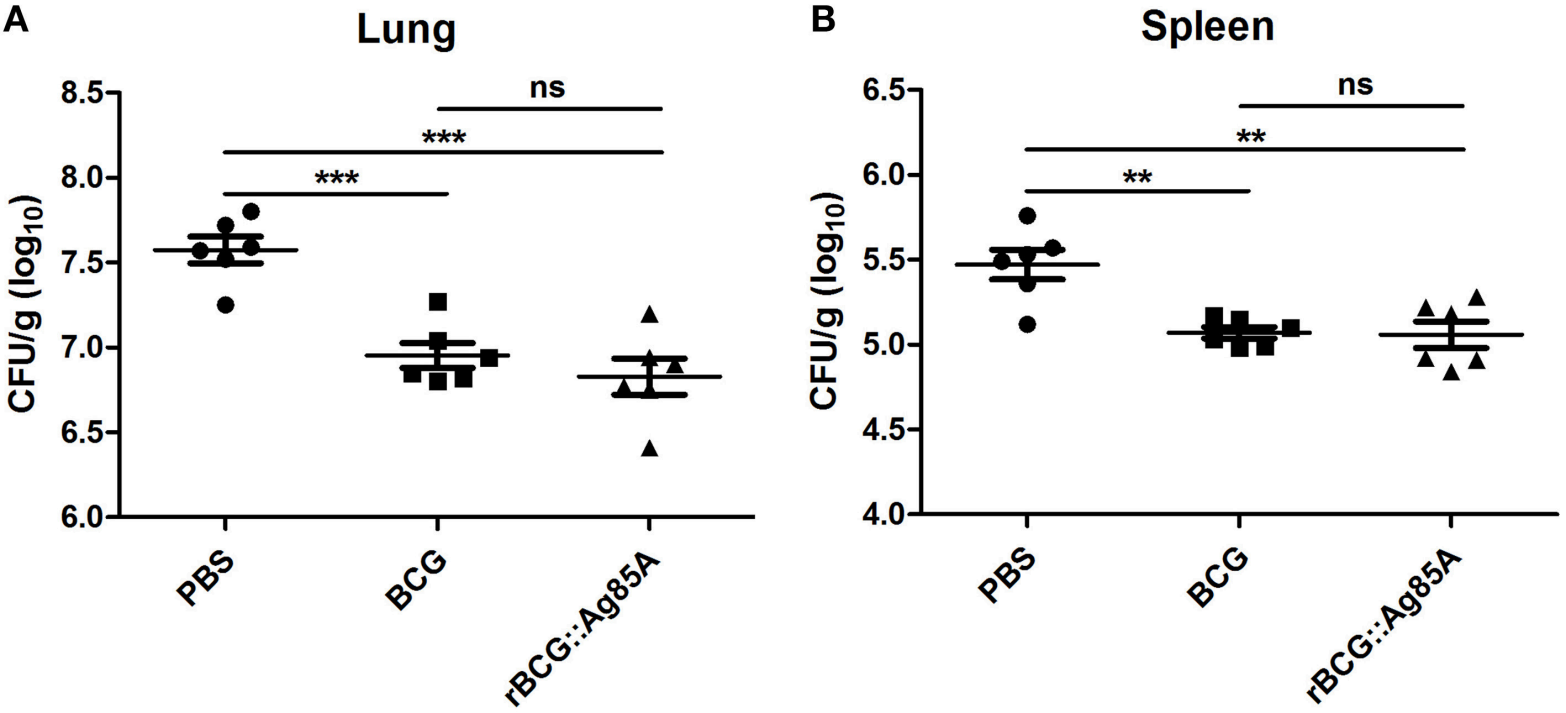
**Bacterial load in the lung and spleen of immunized, TB challenged mice**. Four weeks post-immunization, C57BL/6 mice (*n* = 6) were challenged with 5 × 10^6^ CFU aerosolized virulent *M. tuberculosis* (strain H37Rv). Six weeks after TB challenge, spleens and lungs were harvested and the numbers of CFU per organ were enumerated. TB challenge led to the highest bacterial load in the lung **(A)** and spleen **(B)** in the PBS control. Vaccination with BCG and rBCG::Ag85A strains strongly reduced bacterial load in the lung and spleen in C57BL/6 mice. Data depicted are the mean values ± SEM. Statistical significance was determined by a Student's *t*-test (^**^P < 0.01, ^***^P < 0.001).

**Figure 7:**
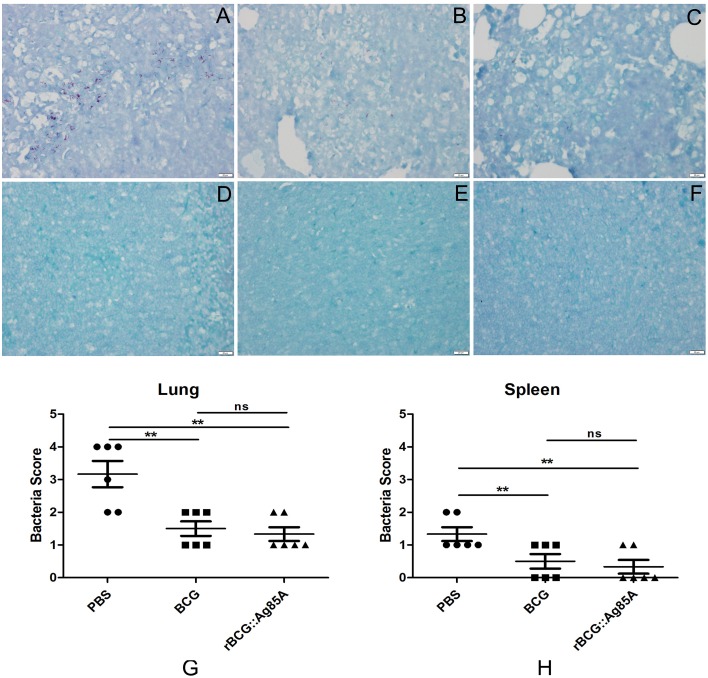
**Histology of the lung and spleen in TB-challenged mice**. Vaccinated C57BL/6 mice were challenged by aerosol with 5 × 10^6^ CFU virulent *M. tuberculosis* H37Rv strain. Six weeks after infection, lung **(A–C,G)** and spleen **(D–F,H)** tissue sections from PBS group **(A,D)**, BCG group **(B,E)**, and rBCG::Ag85A group **(C,F)** were prepared for acid-fast staining (× 4). The number of bacteria in the lung and spleen from the group immunized with rBCG::Ag85A **(C,F)** was lower than PBS group **(A,D)** and BCG group **(B,E)**. Vaccination with BCG and rBCG::Ag85A strains strongly reduced bacterial load in the lung and spleen in C57BL/6 mice **(G,H)**. Data depicted are the mean values ± SEM. Statistical significance was determined by a Student's *t*-test (^**^P < 0.01).

To determine the immune-protective efficacy of the rBCG::Ag85A strain, C57BL/6 mice immunized with rBCG::Ag85A or BCG were challenged with *M. tuberculosis*. 6 weeks post challenge, mice were sacrificed and bacterial load in the lung and spleen of each mouse was analyzed. TB challenge led to the highest bacterial load in the lung and spleen in the PBS control (Figure 6). Vaccination with BCG and rBCG::Ag85A strains strongly reduced bacterial load in the lung and spleen in C57BL/6 mice. rBCG::Ag85A inhibited the growth of *M. tuberculosis* significantly in the lung and spleen compared with PBS group, however, rBCG::Ag85A resulted in higher but not significant decrease of bacterial load compared to BCG vaccinated C57BL/6 mice. These results were confirmed by acid-fast staining histology, which showed that the number of bacteria in the lung and spleen from the group immunized with rBCG::Ag85A was lower than other groups (Figure 7).

### Evaluation of pathological changes

**Figure 8:**
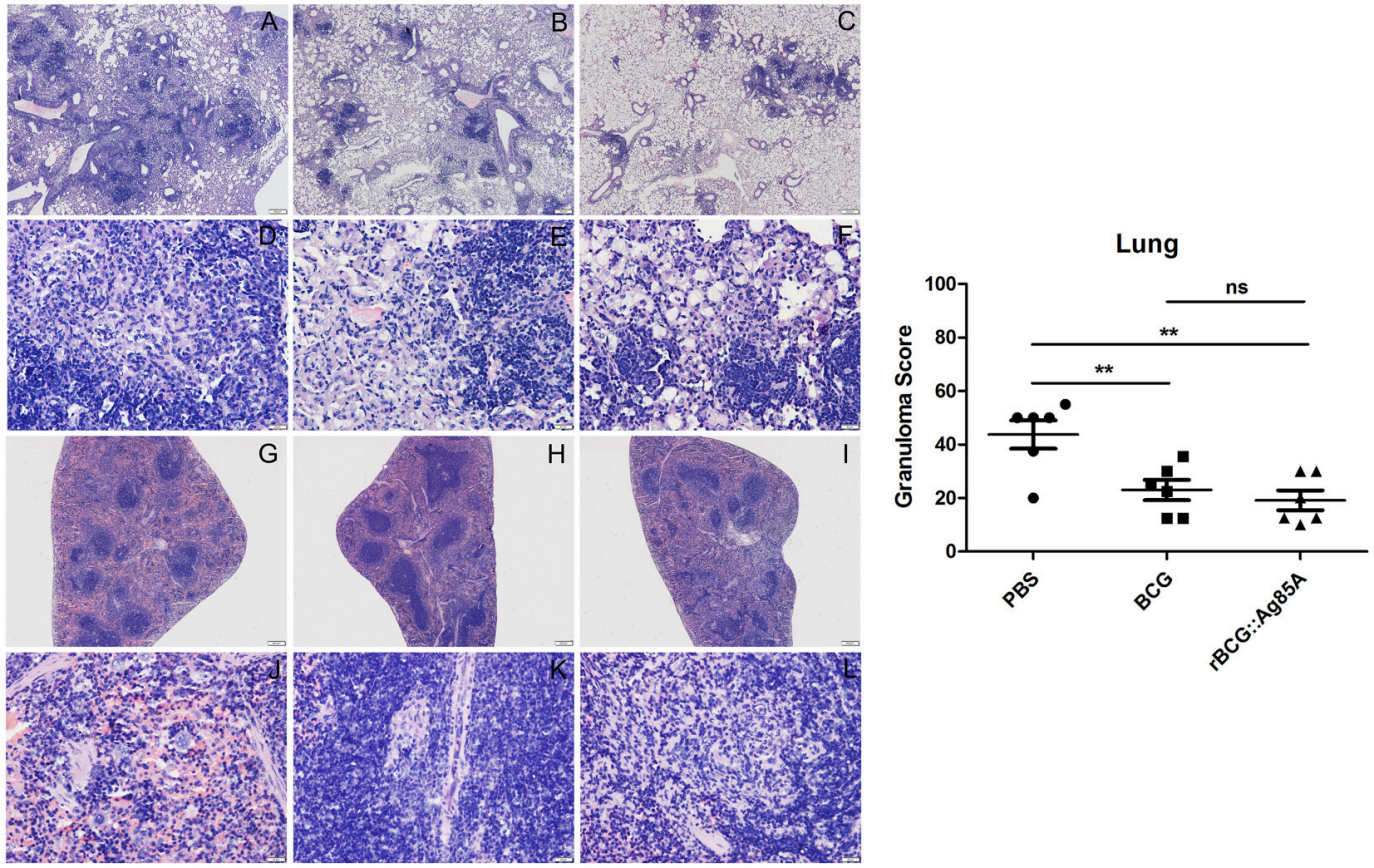
**Representative lung and spleen pathology of C57BL/6 mice after challenge**. Vaccinated C57BL/6 mice were challenged by aerosol with 5 × 10^6^ CFU virulent *M. tuberculosis* H37Rv strain. Six weeks after infection, lung **(A–F)** and spleen **(G–L)** tissue sections from PBS groups **(A,D,G,J)**, BCG group **(B,E,H,K)**, and rBCG::Ag85A group **(C,F,I,L)** were prepared for HE staining (× 160). The most serious pathological changes occurred in the lung of the mice from the PBS control group **(A,D)**, which exhibited the largest number of granuloma-like nodes. Comparatively, pathological changes were less severe in the lung tissue from mice vaccinated with BCG and rBCG::Ag85A **(B,C,E,F)**. No obvious pathological changes were observed in the spleen tissue of mice in any treatment group **(G–L)**. Data depicted are the mean values ± SEM. Statistical significance was determined by a Student's *t*-test (^**^P < 0.01).

To evaluate pathological changes in immunized mice, lung, and spleen tissues from different groups of C57BL/6 mice were fixed, sectioned, and stained with HE stain for assessment of pathological changes. Six weeks after challenge with TB, the most serious pathological changes occurred in the lungs of the mice from the PBS control group, which exhibited the largest number of granuloma-like nodes (Figure 8). Comparatively, pathological changes were less severe in the lung tissue from mice vaccinated with BCG and rBCG::Ag85A, and damage to the lung tissue of rBCG::Ag85A immunized mice was less advanced than in the BCG immunized group. The results of the lung histopathology suggest that rBCG::Ag85A is more effective at protecting lung tissue from damage caused by *M. tuberculosis* infection than BCG. No obvious pathological changes were observed in the spleen tissue of mice in any treatment group.

## Discussion

*M. bovis* BCG is currently the only vaccine available against TB, but it does not prevent the development of pulmonary TB in adults (Fine, 1995; Andersen and Doherty, 2005). As a result, new strategies are needed to improve the effectiveness of the current TB vaccine. As vaccine components, the Ag85 complex proteins have been extensively explored, it has been shown that rBCG overexpressing Ag85B enhanced the immunogenicity of BCG and induced a robust and long-lasting immune response (Xu et al., 2007; Wang et al., 2009; Shen et al., 2010). In the present study, we constructed a recombinant BCG strain overexpressing the immunodominant Ag85A antigen and evaluated its immunogenicity and protective efficacy.

It has been confirmed that antigens expressed, secreted, or linked to the cell membrane by BCG can influence the timing of immune responses and the pathways by which antigen is presented to immune cells (Dennehy and Williamson, 2005). The presentation pathways, for example, via the MHC class II pathway, will determine the cellular and humoral immune responses induced by rBCG. In this study, we explored the antigen presenting kinetics of a recombinant BCG strain overexpressing the immunodominant antigen Ag85A in mice. The Ag85A peptide and MHC complexes were detected on draining lymph node DCs from BCG-infected mice 4 h post-infection with rBCG, and rBCG::Ag85A induced higher levels of Ag85A-specific antigen-presenting activity than those observed for BCG.

CD4^+^ T cells and IFN-γ responses are important components of immune responses against *M. tuberculosis*, and the CD4^+^ T cell response is important in controlling *M. tuberculosis* infection and preventing or delaying the onset of disease (Flynn et al., 1993; Flynn, 2004). We predicted that BCG bacteria producing Ag85A would stimulate IFN-γ production in TB challenged mice better than BCG alone. Lymphocytes isolated from BCG- and rBCG::Ag85A-immunized mice exhibited increased IFN-γ secretion, while mock-immunized mice showed only a slight IFN-γ response. Further, IFN-γ production by lymphocytes from rBCG::Ag85A-immunized mice was significantly higher than in BCG-immunized mice, suggesting that rBCG::Ag85A may induce stronger cellular responses than BCG in mice.

The production of immunoglobulins able to recognize TB epitopes is essential for immune surveillance and rapid response to infection. To determine whether the rBCG::Ag85A strain increases antibody production, antibody titers in the rBCG::Ag85A-immunized mice and BCG-immunized mice were assessed. rBCG::Ag85A immunization generated significantly higher IgG antibody titers than those of the BCG strain, indicating that rBCG::Ag85A stimulates more robust immunoglobulin production than BCG. In mice, the IgG2b antibody response is associated with a Th1-type immune response, whereas the Th2 cytokine response is characterized by IgG1 and IgE antibody responses (Mosmann and Coffman, 1989). The levels of Ag85A-specific IgG1 and IgG2b were examined in the sera of mice infected with rBCG::Ag85A or BCG. IgG2b titer was found to be significantly higher than IgG1 in the rBCG::Ag85A-infected mice, suggesting that rBCG::Ag85A may produce stronger Th1 responses than BCG in mice.

Previous experiments using recombinant BCG strains demonstrated that the insertion of genes encoding immunodominant antigens such as Ag85B, MPT64, ESAT-6, or PPE protein (Xu et al., 2007; Qie et al., 2009; Wang et al., 2009) and immunoregulatory cytokines such as IL-2, IFN-γ, and TNF-α (Murray et al., 1996; Wangoo et al., 2000; Shen et al., 2010) provided more protective efficacy than the parent BCG vaccine. Our recombinant BCG strain rBCG::Ag85A exhibited similar effects: bacterial load in the lung and spleen after TB challenge was decreased by inoculation with rBCG::Ag85A and BCG, and rBCG::Ag85A resulted in a slightly enhanced, although not significant, decrease of bacterial load compared to the BCG vaccination. Results of acid-fast staining of lung tissue from each treatment group showed that the most serious pathological changes of lung occurred in PBS control group, while the rBCG::Ag85A group exhibited the least tissue damage among all groups. These results suggest that the ability of rBCG::Ag85A may be more effective at preventing tissue damage caused by *M. tuberculosis* infection than BCG.

In our immunization experiments, the bacterial loads and pathological changes were all less in the mice immunized with rBCG::Ag85A compared with those induced by BCG immunization, however, the protective efficacy of rBCG::Ag85A was not significantly higher than the parental BCG. As a result, more research is needed to further enhance the expression of recombinant Ag85A protein in rBCG, and to optimize immunization methods and dose administration.

In summary, we have constructed a recombinant BCG strain overexpressing the immunodominant antigen Ag85A and evaluated its immunogenicity and protective efficacy in mice. Our results demonstrate that rBCG::Ag85A improves the immunogenicity of BCG, and induces significantly enhanced cellular and humoral immune responses in mice. Although not statistically significant, mice immunized with the rBCG::Ag85A strain are slightly better protected against *M. tuberculosis* H37Rv infection compared with the BCG vaccine, thus demonstrating its therapeutic potential as a vaccine against TB. However, further improvement and evaluation of recombinant BCG are necessary.

## Author contributions

ZX, XC, and XJ designed the experiments. ZX, TH, CM, XW, and YR performed the experiments and analyzed the data. XC, YY, ZP, and XJ contributed reagents/materials/analysis tools. ZX, XC, XZ, and XJ wrote and revised the paper.

### Conflict of interest statement

The authors declare that the research was conducted in the absence of any commercial or financial relationships that could be construed as a potential conflict of interest.

## Supplementary material

Figure S1 **Construction and verification of recombinant BCG**. M1: DL2000 DNA ladder; 1: Amplification of the *fbpA* gene **(A)**. M1: λ-EcoT14 DNA ladder; M2: DL2000 DNA ladder; 1: pMV261–*fbpA* digested with *Bam*H Iand *Eco*RI **(B)**. M1: DL2000 DNA ladder; 1: BCG confirmed by PCR, the primers bind to the vector which is not present in wild type BCG; 2: rBCG::Ag85A confirmed by PCR, the primers bind to the vector which is present in recombinant BCG **(C)**.
