# Supplementary figures and images for: Evaluation of Immunogenicity and Protective Efficacy Elicited by Mycobacterium bovis BCG Overexpressing Ag85A Protein against Mycobacterium tuberculosis Aerosol Infection
Source: Front Cell Infect Microbiol. 2016 Jan 28;6:3. doi: 10.3389/fcimb.2016.00003 (PMC4729882; doi:10.3389/fcimb.2016.00003)

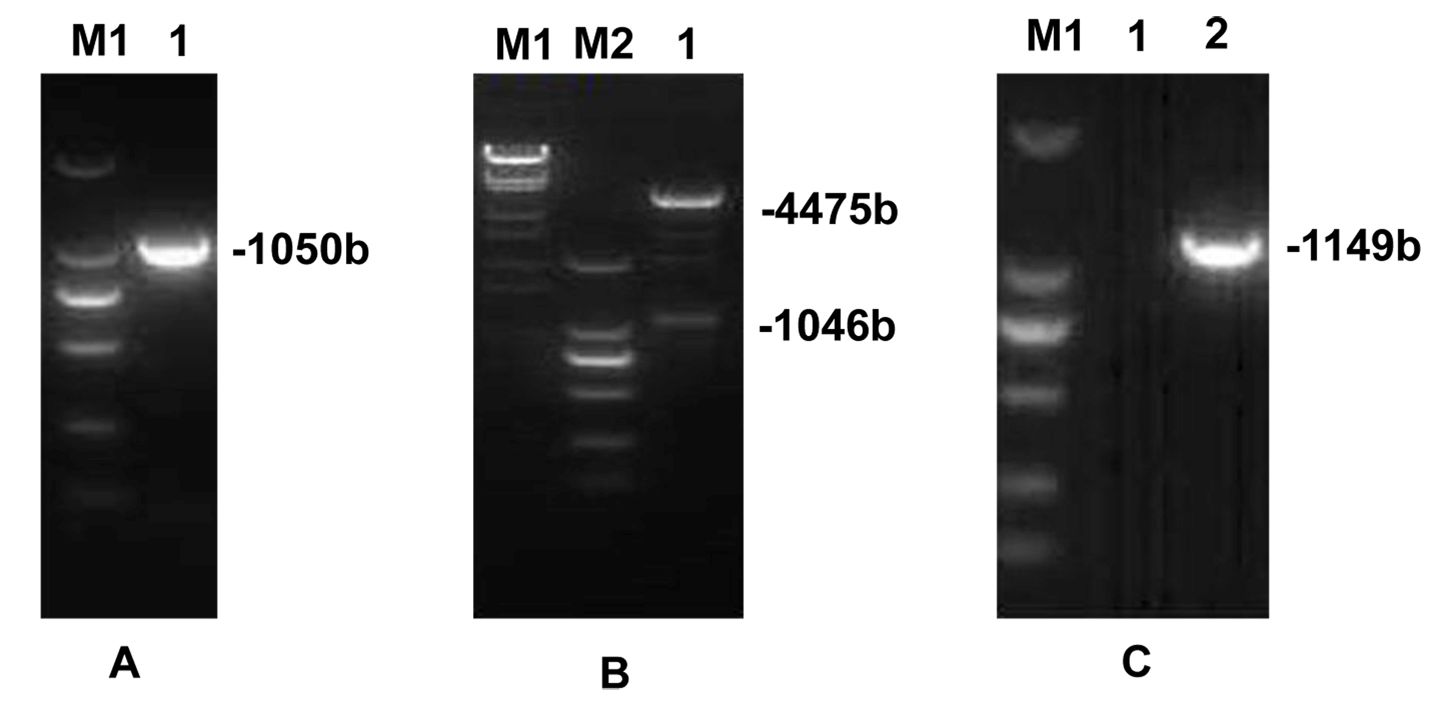

Supplement: Figure S1 — Construction and verification of recombinant BCG. M1: DL2000 DNA ladder; 1: Amplification of the fbpA gene (A). M1: λ-EcoT14 DNA ladder; M2: DL2000 DNA ladder; 1: pMV261–fbpA digested with BamH Iand EcoRI (B). M1: DL2000 DNA ladder; 1: BCG confirmed by PCR, the primers bind to the vector which is not present in wild type BCG; 2: rBCG::Ag85A confirmed by PCR, the primers bind to the vector which is present in recombinant BCG (C). [file Image1.JPEG]
